# Passive Brain-Computer Interfaces for Enhanced Human-Robot Interaction

**DOI:** 10.3389/frobt.2020.00125

**Published:** 2020-10-02

**Authors:** Maryam Alimardani, Kazuo Hiraki

**Affiliations:** ^1^Department of Cognitive Science and Artificial Intelligence, School of Humanities and Digital Sciences, Tilburg University, Tilburg, Netherlands; ^2^Department of General Systems Studies, Graduate School of Arts and Sciences, The University of Tokyo, Tokyo, Japan

**Keywords:** brain-computer interface (BCI), passive BCIs, human-robot interaction (HRI), cognitive workload estimation, error detection, emotion recognition, EEG, social robots

## Abstract

Brain-computer interfaces (BCIs) have long been seen as control interfaces that translate changes in brain activity, produced either by means of a volitional modulation or in response to an external stimulation. However, recent trends in the BCI and neurofeedback research highlight passive monitoring of a user's brain activity in order to estimate cognitive load, attention level, perceived errors and emotions. Extraction of such higher order information from brain signals is seen as a gateway for facilitation of interaction between humans and intelligent systems. Particularly in the field of robotics, passive BCIs provide a promising channel for prediction of user's cognitive and affective state for development of a user-adaptive interaction. In this paper, we first illustrate the state of the art in passive BCI technology and then provide examples of BCI employment in human-robot interaction (HRI). We finally discuss the prospects and challenges in integration of passive BCIs in socially demanding HRI settings. This work intends to inform HRI community of the opportunities offered by passive BCI systems for enhancement of human-robot interaction while recognizing potential pitfalls.

## Introduction

For generations, the idea of having intelligent machines that can read people's minds and react without direct communication had captured human's imagination. With recent advances in neuroimaging technologies and brain-computer interfaces (BCI), such images are finally turning into reality (Nam et al., 2018). BCIs are the systems that decode brain activity into meaningful commands for machines, thereby bridging the human brain and the outside world. BCIs are primarily developed as a non-muscular communication and control channel for patients suffering from severe motor impairments (Millán et al., 2010; Chaudhary et al., 2015; Lebedev and Nicolelis, 2017; Chen et al., 2019). For instance, a BCI-actuated wheelchair or exoskeleton can assist a patient with ALS or spinal cord injury to regain mobility (Kim et al., 2016; Benabid et al., 2019). Similarly, locked-in patients can be equipped with a BCI system in order to effectively communicate with external world (Sellers et al., 2014; Hong et al., 2018; Birbaumer and Rana, 2019). Stroke patients have also demonstrated effective restoration of motor functions and improvement of life quality after they were trained with a BCI-control task in a neurological rehabilitation session (Soekadar et al., 2015).

However, with the growing popularity of BCIs, new application corners outside of the medical field have emerged for healthy users (Allison et al., 2012; Van Erp et al., 2012; Nam et al., 2018). One of the mainstream applications is the integration of BCIs with other interactive technologies such as virtual reality (VR) and computer games (Lécuyer et al., 2008; Coogan and He, 2018). Several prototypes have already been developed that enable a user to either navigate through a virtual space or manipulate a digital object only by means of thoughts (Friedman, 2015). The combination of immersive technologies and BCIs entails a two-way benefit for researchers in that the BCI system provides a new form of control channel over the environment, thus changing the user experience, and virtual environments serve as a suitable platform for BCI research as they offer a safe, engaging, and cost-effective tool for the design of BCI experiments and neurofeedback (Allison et al., 2012; Lotte et al., 2012).

In addition to immersive environments, BCIs have also been utilized in combination with physical robots in order to induce a sense of robotic embodiment and remote presence (Alimardani et al., 2013; Beraldo et al., 2018). In these setups, users control a humanoid robotic body and navigate through the physical space by means of their brain activity while they can see through the robot's eyes. Such interactions often lead to a feeling of telepresence and the experience of losing boundary between the real body and the robot's body (Alimardani et al., 2015), paving the way for research in cognitive neuroscience and neural prosthetics (Pazzaglia and Molinari, 2016).

In all the above-mentioned examples, the brain activity features extracted for the BCI classifier are either voluntarily induced by the user (active control) or measured as a response to an external stimulus (reactive control). Such BCI systems that require users to get involved in a cognitive task and provide explicit commands are referred to as active BCIs (Zander and Kothe, 2011; Lightbody et al., 2014). On the other hand, BCIs that are event driven and measure brain responses to a visual, auditory or haptic stimulus are called reactive BCIs (Zander and Kothe, 2011). However, there is a third group of BCIs that drive their outputs from spontaneous brain activity without the need from the user to perform specific mental tasks or receive stimuli. These BCI systems, which normally monitor longer epochs of brain activity for detection of a cognitive state change or emotional arousal, are called passive BCIs (Zander and Kothe, 2011; Aricò et al., 2018). An example of this is a system that monitors a driver's neural dynamics in real-time and alarms him/her in the case of drowsiness detection (Lin C. T. et al., 2010; Khan and Hong, 2015).

Passive BCIs primarily aim at detecting unintentional changes in a user's cognitive state as an input for other adaptive systems (Zander et al., 2010; Aricò et al., 2016). For instance, in the driving example, the output of the BCI system that evaluates driver drowsiness can alternatively be used for administration of the temperature in the car or the volume of the sound system in order to increase alertness of the driver (Liu et al., 2013). Similarly, a BCI that extracts information about a user's ongoing cognitive load and affective states offers numerous applications in the design of adaptive systems and social agents that would adjust their behavior to the user's ongoing mental state, without distracting the user from the main task, thereby enriching the quality of interaction and performance (Szafir and Mutlu, 2012; Alimardani and Hiraki, 2017; Zander et al., 2017; Ehrlich and Cheng, 2018).

In this article, we mainly discuss passive BCIs in the context of human-computer and human-robot interaction. In section BCIs and Cognitive/Affective State Estimation, we first lay out the state of the art in passive BCIs by briefly reviewing existing studies that attempted detection of cognitive and affective state changes from brain responses. We restricted our literature search to studies that adopted electroencephalography (EEG) signals for development of the BCI classifier. Given its mobility, high temporal resolution, and relatively low price, EEG is considered as a feasible non-invasive brain imaging technique that can be deployed into a wide variety of applications including human-robot interaction. In section BCIs and Human-Robot Interaction, we focus on passive BCI-robot studies that used cognitive and affective state measures as a neurofeedback input for a social or mechanical robot, thereby optimizing their response and behavior in a closed-loop interaction. In the last section, we discuss the prospects and challenges that are faced in the employment of passive BCIs in real-world human-robot interaction.

## BCIs and Cognitive/Affective State Estimation

In neuroscientific literature, cognitive state estimation refers to the quantification of neurophysiological processes that underlie attention, working-memory load, perception, reasoning, and decision-making, while affective computing targets assessment of the emotional experience. BCI systems that decode covert information in the brain signals regarding these internal processes can establish an implicit communication channel for an adaptive human-technology interaction, presenting novel applications in the domains of education, entertainment, healthcare, marketing, etc. (Van Erp et al., 2012; Blankertz et al., 2016; Krol and Zander, 2017; Aricò et al., 2018). We identified three main directions for assessment of cognitive and affective states in EEG-based passive BCIs; (1) detection of attention and mental fatigue, (2) detection of errors, and (3) detection of emotions. In the following, we describe the current state of the art in each of these domains, laying out a foundation for future employment of passive BCIs in human-robot interaction.

### Detection of Attention and Mental Fatigue

As discussed in the drowsy driver example, monitoring real-time mental workload and vigilance is of particular importance in safety-critical environments (Lin C. T. et al., 2010; Khan and Hong, 2015; Aricò et al., 2017). Non-invasive BCIs that detect drops in attention level and increased mental fatigue can be utilized in a broad range of operational environments and application domains including aviation (Aricò et al., 2016; Hou et al., 2017) and industrial workspaces (Schultze-Kraft et al., 2012) where safety and efficiency are important, as well as educational and healthcare setups where the system can provide feedback from learners to a teacher (Ko et al., 2017; Spüler et al., 2017), evaluate sustained attention in e-learning platforms (Chen et al., 2017), and execute attention training for clinical patients who suffer from attention deficit hyperactivity disorder (ADHD) (Lim et al., 2019). It is even suggested that detection of attention level can be employed in a hybrid BCI system in which an attention classifier is integrated with other BCI algorithms in order to confirm users' focus on the BCI task and validate the produced response, thereby yielding a more reliable and robust performance (Diez et al., 2015).

Multiple algorithms have already been proposed to quantify the level of alertness and mental workload within EEG brain activity. A large number of these models rely on frequency domain features such as theta, alpha and beta band powers, for estimation of attention level and mental fatigue experienced by the user (Lin C. T. et al., 2010; Roy et al., 2013; Diez et al., 2015; Khan and Hong, 2015; Aricò et al., 2016; Lim et al., 2019). On the other hand, some studies have examined non-linear complexity measures of time series EEG signals such as entropy (Liu et al., 2010; Min et al., 2017; Mu et al., 2017), promoting a fast and less costly method for real-time processing. Although not very common, a few studies have also proposed the usage of event-related potentials (ERP), such as non-target P300, in development of passive classifiers given that such brain responses are affected by both attention and fatigue and thus can provide a measure of target recognition processes (Kirchner et al., 2013; McDaniel et al., 2018).

In addition to spectral and temporal information carried by EEG signals, spatial features such as brain regions from which the signals were collected have been shown important in the detection of different mental state changes (Myrden and Chau, 2017). Although reported results are not always consistent, there is a general consensus on the role of frontal lobe in discrimination of cognitive workload and task difficulty (Zarjam et al., 2015; Dimitrakopoulos et al., 2017), prefrontal and central lobes in detection of fatigue and drowsiness (Min et al., 2017; Ogino and Mitsukura, 2018), and posterior areas (particularly posterior alpha band) in estimation of visuospatial attention (Ko et al., 2017; Myrden and Chau, 2017). It is worth noting that functional connectivity between different brain regions is also suggested in the literature as an index for estimation of engagement and attention (Dimitriadis et al., 2015; Dimitrakopoulos et al., 2017), although due to computational cost it poses limitations on real-time implementation.

### Detection of Errors

Failures during technology usage and outputs that deviate from expectation can become a source of dissatisfaction and additional cognitive workload for the user. Unintentional mistakes made by the human or erroneous behavior presented by the system can generate user frustration and aggravate human-system interaction (Zander et al., 2010). Such negative repercussions can be prevented by automatic detection and feedback of errors, as perceived by the user, for online correction or adaptation of system characteristics while the user is still involved in the interaction (Zander et al., 2010; Chavarriaga et al., 2014; Krol and Zander, 2017).

When a user recognizes a mismatch from expectation, an error-related potential (ErrP) is generated in the EEG signals. A passive BCI system that extracts this information in real-time can be used in development of hybrid and adaptive systems that optimize the performance of the user either by removing the erroneous trials (Ferrez and Millán, 2008; Schmidt et al., 2012; Yousefi et al., 2019), or by modifying the classification parameters through online learning of the BCI classifier (Krol and Zander, 2017; Mousavi and de Sa, 2019), or by adjusting the task difficulty level to different individuals in order to improve engagement and motivation (Mattout et al., 2015). For instance, Ferrez and Millán (2008) combined a motor imagery BCI with an error detection algorithm that looked for an ErrP immediately after each trial and filtered out trials that contained an error-related response. Their results displayed a significant improvement of BCI performance in real-time by reducing the classification error rate from 30 to 7%. Similarly, Schmidt et al. (2012) combined online detection of ErrPs with a BCI speller and reported 49% improvement in the mean spelling speed. In a recent report, Dehais et al. (2019) presented a passive BCI classifier for prediction of auditory attentional errors during a real flight condition, proposing future smart cockpits that would adapt to pilots' cognitive needs.

A unique feature of ErrPs is that they would arise in response to any form of discrepancy during interaction/task execution including when the user realizes a self-made error (response ErrP), when s/he is informed about the error through some type of feedback (feedback ErrP), and even when the user senses an error made by a third party (observation ErrP) (Ferrez and Millán, 2005; Gürkök and Nijholt, 2012; Vi et al., 2014). This permits detection and management of errors in any form and at any time during the interaction, promoting closed-loop passive BCIs not only as an efficient and seamless tool for online evaluation of user performance but also as a secondary communication tool in multi-user collaborative environments such as emergency rooms (Vi et al., 2014) where agile and high-risk decision making is required (Poli et al., 2014).

Additionally, recent efforts suggest that different kinds of errors generate different ErrPs, allowing discrimination of error severity and error types (Spüler and Niethammer, 2015; Wirth et al., 2019) based on temporal, spectral, and spatial information in the EEG waveforms. However, the downside of this approach is that, in most cases, the ErrP classifier relies on an event-locked paradigm in which ErrPs can only be extracted within a fixed window from a specified trigger. In real-world applications, the information regarding stimulus time or origin of the error is often unavailable and the latency of user responses may vary across individuals and tasks. Therefore, future integration of such passive BCIs with natural human-agent interactions calls for further developments on self-paced algorithms that make asynchronous error detection possible at any time during the interaction (Lightbody et al., 2014; Spüler and Niethammer, 2015; Yousefi et al., 2019).

### Detection of Emotions

With advancement of commercially available wearable sensors, estimation of human emotions from ongoing biosignals has received increased attention in recent years (Al-Nafjan et al., 2017; Shu et al., 2018; García-Martínez et al., 2019; Dzedzickis et al., 2020). Emotions are particularly important in the design of intelligent and socially interactive systems as they enable the digital agents to generate a well-suited behavior and establish an affective loop with the human partner (Paiva et al., 2014; Ehrlich et al., 2017). Compared to conventional methods of social signal processing and affective computing (such as voice and image processing), biosignals present the advantage of containing spontaneous and less controllable features of emotions. Emotions entail three aspects; physiological arousal, conscious experience of the emotion (subjective feeling) and behavioral expression (Alarcao and Fonseca, 2017). Voice and face recognition technologies can only capture the third aspect, i.e., overt behavioral expression of emotion, whereas brain activity can inform us about the neurophysiological and cognitive processes that generate and lead to such emotional states (Mühl et al., 2014a).

A major challenge in classification of emotions from brain activity is that there is not a unique computational method for extraction and mapping of emotion-related features. There are two theories in the modeling of emotions; discrete model and dimensional model (Kim et al., 2013). The former defines emotions as a set of categorical affective states that represent core emotions such as happiness, sadness, anger, disgust, fear, and surprise (Lin Y. P. et al., 2010; Jenke et al., 2014). The latter maps emotions on either a two-dimensional valence-arousal space (Posner et al., 2005; Atkinson and Campos, 2016) or a three-dimensional valence-arousal-dominance space (Mehrabian, 1996; Reuderink et al., 2013). The discrete model is more popular among BCI developers as it reduces the problem of dimensionality, however it does not consider that the same emotion may manifest on different scales of arousal, valence and dominance. The dimensional model provides continuity as it quantifies emotions on each dimension (valence ranging from positive to negative, arousal ranging from calm to excited and dominance ranging from in-control to submission). Particularly, the 2D model has been previously used in multiple EEG studies (Liberati et al., 2015; Al-Nafjan et al., 2017; Mohammadi et al., 2017), however in these studies, the dimensionality is often simplified again by means of clustering emotions across the valence-arousal coordinates (e.g., fear as negative valence, high arousal or happiness as positive valence, high arousal), which bears the risk of grouping different emotions that share the same valence and arousal levels (e.g., anger and fear) in one cluster (Liberati et al., 2015).

Another challenge in the development of emotional BCIs is the diverse elicitation strategies that exist in the affective computing literature. Multiple types of stimuli including affective pictures, sounds, video fragments and music have been used in the past in order to induce emotional responses (Al-Nafjan et al., 2017). In addition to the lack of consistency among reported results and available EEG datasets, an inherent problem with these forms of stimuli is that there is no evidence whether the induced emotion is a natural affective state or just a reactive response to the stimulus. To counter this issue, some studies have employed a self-induced strategy such as recall of autobiographical emotional memory (Chanel et al., 2009; Iacoviello et al., 2015) or imagination of the emotion by means of verbal narratives (Kothe et al., 2013). This method entails other problems; the self-induced emotions are inevitably weaker than those induced by external stimuli, and users are prone to distraction during the task as it is difficult to maintain mental imageries for a long period (Chanel et al., 2009).

It is worth mentioning that emotions are more than just an affective state for social interaction and adaptive environments; they may also influence other cognitive functions. For instance, frustration can extend negative impacts on attention, decision-making, learning, and response accuracy. Indeed, past research has shown that affective states such as stress, anxiety and frustration can influence BCI performance in estimation of mental workload and attention (Mühl et al., 2014b; Myrden and Chau, 2015; Lotte et al., 2018). Thus, it can be expected that an adaptive multimodal BCI system that identifies users' affective states and regulates tasks accordingly would improve user performance and validity of the system in the long term (Gürkök and Nijholt, 2012).

To sum up, there have been several BCI algorithms proposed for detection of affective state changes from EEG signals (Alarcao and Fonseca, 2017), however, automatic recognition of emotions during ecologically valid tasks and natural interactions remains a challenge, hindering deployment of affective BCIs in other platforms such as human-robot interaction. Future research should attend currently existing issues such as insufficient classification accuracy, inconsistent computational and elicitation techniques, as well as development of BCI models that can extract emotions in an unobtrusive and asynchronous manner over a long period of time.

## BCIs and Human-Robot Interaction

**Figure 1:**
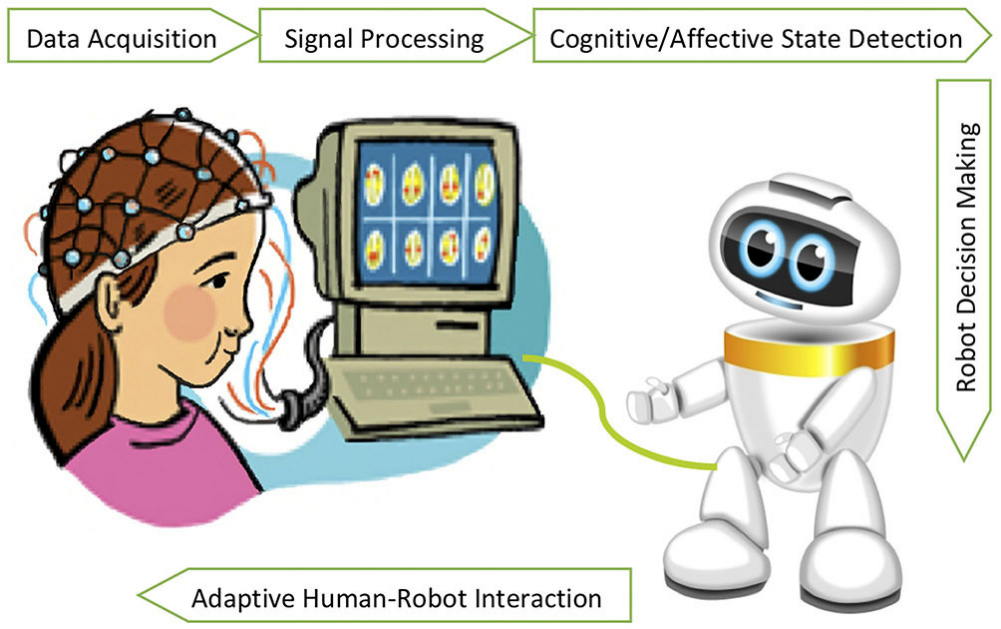
Closed-loop human-robot interaction using passive BCIs.

With more integration of robots into our daily life, the necessity for them to function as social and assistive companions in real-world environments such as schools and healthcare facilities becomes eminent. In addition to human's intentions and control commands, it is crucial for the robots to estimate the emotional states of a human partner in order to be socially responsive, engage longer with users and promote natural HRI (Ficocelli et al., 2015). More importantly, estimation of workload, anxiety and errors is crucial for ergonomic and safe human-robot collaboration in both domestic and industrial spaces (Ajoudani et al., 2018). In this section, we particularly discuss studies that have employed BCIs for passive detection of cognitive and affective states of a human user in order to effectively adapt the behavior of a robot in a closed-loop interaction with the human partner (Figure 1).

We restricted our literature search to only non-invasive BCI studies that passively extracted user's cognitive and affective states during interaction with a physical robot, therefore, articles that employed active BCIs for motion control (e.g., motor-imagery based robot operation) or reactive BCIs for intentional selection of behavior for a robotic interface (e.g., robot manipulation triggered by event-related P300 or Steady State Visually Evoked Potential SSVEP) were not included. Another inclusion criterion was usage of AI-powered predictive models together with EEG signals in the study, where a passive BCI classifier was used (or its development was attempted) during real-time interaction with a robot. Neuroscience research in which only brain oscillation patterns associated with robot interaction are reported were either excluded or already reported in section BCIs and Cognitive/Affective State Estimation. Finally, the study should have reported a passive BCI interaction with a physical robot; interactions with virtual or simulated agents were excluded as the definition of a simulated agent is very board and incorporates human-computer interaction and game applications of passive BCIs. The inclusion and exclusion criteria defined for review of BCI-HRI studies in this section are summarized in Table 1.

**Table 1:** The inclusion and exclusion criteria as used for the selection of BCI-HRI studies in section BCIs and Human-Robot Interaction.

**Parameters**	**Inclusion criteria**	**Exclusion criteria**
Type of BCI	Passive BCIs (hybrid with other BCI types acceptable)	Active or reactive BCIs (e.g., motor imagery, ERP, SSVEP)
Type of signal	EEG (hybrid with other signal types acceptable)	fNIRS, fMRI, MEG
Type of interaction	Interaction with physical robots (e.g., social robots, arm robots)	Interaction with virtual avatars, computer games
Type of analysis	Real-time classification/ feedback to the robot	Offline analysis of brain signals captured during HRI

Our search resulted in a total of 10 studies as shown in Table 2. In the following, we briefly describe the methodology and outcomes of each listed study.

**Table 2:** List of articles in the literature that used a passive BCI classifier for extraction of user's cognitive and affective state during interaction with a physical robot.

**References**	**EEG feature**	**BCI classifier output**	**Adaptive HRI**
Szafir and Mutlu (2012)	Spectral band powers	Attention drops in user during storytelling by a robot	The robot provided attention-evoking cues
Kirchner et al. (2013)	Absence of P300	Stimuli recognition during teleoperation of an exoskeleton arm	The robot controller repeated stimuli or changed response window if the user missed the stimuli
Ehrlich et al. (2014)	Spectral band powers	User intention to initiate eye-contact with a robot	None
Iturrate et al. (2015)	Error-related potential	Erroneous motor behavior by an artificial robotic arm in a reaching task	The robot arm controller learned correct and incorrect behavior through reinforcement learning
Kim et al. (2017)	Error-related potential	Wrong mapping between user's gestures and robot's action	The robot updated action-selection strategy and learned gesture meaning through reinforcement learning
Salazar-Gomez et al. (2017)	Error-related potential	Erroneous robot motion in a binary reaching task	The robot switched trajectory based on the observer's EEG response
Ehrlich and Cheng (2018)	Error-related potential	Mismatch in gaze behavior	The robot adapted gaze behavior based on decoded ErrPs
Ehrlich and Cheng (2019)	Error-related potential	Erroneous robot head movement as a response to a directional key press by the user	None
Shao et al. (2019)	Spectral asymmetry	Emotional valence (positive vs. negative) during exercise with a robot coach	The robot provided verbal and non-verbal feedback based on the user's affect and engagement level
Lopes-Dias et al. (2019)	Error-related potential	Erroneous arm robot movement when the robot should have imitated human hand movement.	The robot would give control to human again to correct his/her movement

Szafir and Mutlu (2012) reported an interesting study in which a humanoid robot monitored students' EEG signals during storytelling and gave them attention-evoking immediacy cues (either in verbal or non-verbal form) whenever engagement drops were detected. In doing so, they extracted EEG levels in alpha, beta and theta frequency bands and smoothed them into an engagement signal that would represent attention levels. Every time the attention level went below a pre-defined threshold, the robot displayed immediacy cues such as increased spoken volume, increased eye contact, and head-nodding. Their results showed that participants who experienced interaction with an adaptive BCI-driven robot had a significantly better recall of the story details than those who participated in an interaction with randomly presented immediacy cues. In addition to this, female participants reported a more favorable evaluation of the robot behavior, in terms of improved motivation and rapport, in the BCI condition compared to the random condition. The results of this study highlight the benefits of BCIs in interactive educational setups where real-time detection of user disengagement and attention drop can be compensated by means of an embodied social agent.

Kirchner et al. (2013) employed passive classification of event-related potential P300 in an adaptive human-robot interaction. They reported a brain reading (BR) system that implicitly extracted p300 during teleoperation of an exoskeleton arm whenever an important stimulus was presented to the user. They used the evoked potential amplitude as an indicator of successful stimuli recognition by the user. If the response did not contain P300 or the potential was not strong enough, it implied that the user had missed the important information that was presented and thus the system repeated the stimuli. Authors found a reduced stress level in subjects when BR was embedded in the control interface, recommending their approach as a promising way to improve the functionality of interactive technical systems.

Ehrlich et al. (2014) proposed an EEG-based framework for detection of social cues such as gaze by a humanoid robot as a measure for social engagement. They instructed subjects to either wait for the robot to make eye-contact with them or to intentionally generate brain patterns for the robot to initiate eye-contact with them (influence the robot's behavior). By extracting frequency band powers as discriminating features in an offline analysis, they could find high classification performance between the two conditions. Such predictive model could be implemented in a human-robot interaction in order to enable the robot to estimate its social role and adapt its behavior to the expectations of the human partner.

Iturrate et al. (2015) introduced a reinforcement learning (RL) algorithm that learned optimal motor behavior of a robotic arm based on observation ErrPs carried in the brain signals of a human viewer. The BCI classifier decoded reaching actions as erroneous whenever ErrPs were present. The non-ErrP trials were then employed as an online reward for the RL algorithm. Their approach improved the number of learned actions and control policies compared to random rewards. Authors suggest their algorithm for future application in neuroprosthetics in which implicit input from the patient can optimize the behavior of an artificial limb for goal-oriented movements.

Kim et al. (2017) conducted a study similar to Iturrate et al. (2015) in which they trained a RL algorithm based on the user's ErrPs in a gesture recognition task. They prepared two scenarios; (1) when a simulated arm robot recognized and copied user's gestures, and (2) when a real arm robot recognized and copied user's gestures. In both scenarios, the ErrP classifier used the correct mappings as a reward for the RL algorithm. They showed that both simulated and real robots could effectively learn gestures from the human instructor with a high online ErrP detection accuracy (90 and 91%, respectively). However, not surprisingly, the learning curve was different across subjects based on the performance of ErrP classifier. Past studies have shown that ERP-based BCI performance varies across individuals based on psycho-cognitive parameters (Sprague et al., 2016) suggesting that ErrP-based BCIs may require subject-specific calibration and training when integrated within an HRI setting.

Salazar-Gomez et al. (2017) introduced a closed-loop control interface for automatic correction of reaching behavior of a robotic arm. They recorded EEG signals from a human observer while the robot was performing a binary object selection task after a cue presentation. They used ErrP responses as a real-time feedback for the robot to switch trajectory if the selected choice was not compatible with the cue. Despite the sound methodology of this study, authors only reported classification results from four subjects, which makes it difficult to draw firm conclusions. Also, no reports were made regarding user perception of the interaction and attitude toward the robot in open-loop vs. closed-loop HRI. However, an interesting finding in this study was the presence of a secondary ErrP in the closed-loop interaction when the human observed an incorrect interpretation of the feedback by the robot (robot not obeying the human or switching to the wrong trajectory due to misclassification). This suggests the design of new BCI paradigms where secondary and further ErrPs can be incorporated in continuous interactions until an optimal behavior is achieved (Cruz et al., 2018).

Ehrlich and Cheng (2018, 2019) reported two consecutive studies in which they used ErrP signals for detection of mismatch between user's intended gaze and actual robot's gaze (Ehrlich and Cheng, 2018) and user's intended head movement and actual robot's head movement (Ehrlich and Cheng, 2019). In the former study, they used a closed-loop interaction (adaptive behavior by the robot) where the user first guessed the direction of robot gaze from three available choices and then the robot performed a random gaze behavior which was followed by an updated behavior based on the ErrP classifier outcome. Using a learning paradigm for the robot's gaze policy, they showed that a mutual adaptation between the human and robot's behavior emerged, leading to a relatively high classification performance and more efficient interaction. In the latter study (Ehrlich and Cheng, 2019), authors again used a guessing game to compare the observability and decodeability of ErrP responses to two experimental stimuli; an incongruent robot movement vs. an incongruent curser movement. In the first condition, participants guessed the robot head movement from three possible directions (left, right, up) using arrow key-presses, and watched the robot perform a random action. In the second condition, they again guessed a possible direction but watched a curser moving either toward or away from that direction on a computer screen. Although they found a satisfactory classification accuracy (69%) in the HRI scenario, they observed that the classification accuracy for ErrP responses was significantly higher in the cursor scenario (90%), indicating more sensitivity of ErrPs to visually simple cues compared to contextual robot actions.

Shao et al. (2019) used a low-cost EEG (InteraXon Muse 2016) together with heart rate and motion sensors during interaction with a health coach humanoid robot. They extracted EEG frequency band powers in order to classify the emotional valence of the user during exercise with the robot. The robot then presented an online positive or negative feedback (happy, interested, worried, and sad) based on the user's affect (positive or negative) and engagement level (engaged or not engaged). The participants of their study reported a high acceptance and perceived usability for the robot, however the robot was tested in a non-controlled experiment (no other condition was compared with the above scenario) and the classification results for the affect recognition model was not particularly high (71%), therefore it is possible that the reported results were merely due to the novelty effect caused by the robot presence, and not emotional awareness and adaptive feedback during the interaction.

Finally, Lopes-Dias et al. (2019) attempted asynchronous decoding of ErrPs during online control of an arm robot. Participants had to move their own hand according to a binary stimuli on the screen and using a motion capture system, the robot was expected to copy the same movement in the physical world. In case the hand movement was not detected correctly, an ErrP signal was detected and the robot allowed the user to correct the error. The major finding of this study was the possibility of asynchronous detection of ErrPs using a sliding window during online robot operation. Authors do not discuss their results in the context of human-robot interaction and a possible embodiment effect (Alimardani et al., 2013), however similar to Salazar-Gomez et al. (2017), they observed secondary ErrPs in some participants which confirms the applicability of these later potentials in improvement of robot performance.

Although, this section only focused on EEG-based passive BCIs for the purpose of HRI, it is worth mentioning the potential of other brain imaging techniques such as fNIRS (Canning and Scheutz, 2013) in passive evaluation of user responses during robot interaction, for instance, detection of cognitive workload during multitasking with two robots (Solovey et al., 2012) or detection of affinity and eeriness in robot appearance (Strait and Scheutz, 2014). Additionally, insights can be driven from passive BCI studies with simulated agents and teleoperated robots (Esfahani and Sundararajan, 2011; Cavazza et al., 2015; Aranyi et al., 2016; Zander et al., 2017) to further inform the HRI community of the possible exploitation avenues.

Altogether, passive BCIs show promise in the design of optimal robot behavior by means of indirect communication from the human partner. Our literature review shows that detection of erroneous robot behavior using ErrP signals is the most popular paradigm for integration of passive BCIs in HRI settings. Contrary to our expectation, there were very few studies that employed detection of mental workload or emotions for adaptive social behavior in HRI. This confirms that despite the great effort of AI community in developing several classification models for EEG-based emotion and cognitive state prediction, real-time incorporation of these models in a closed-loop interaction with physical robots are yet not adequately explored. This gap should be addressed by BCI and HRI researchers in the future, thereby creating a synergy between the two domains for promotion of socially intelligent and adaptive robots.

## Prospects and Challenges

As discussed in previous sections, passive BCIs offer a promising means to objectively monitor cognitive and affective states of a technology user either as an offline evaluation metric of the user's performance or as a communication modality for closed-loop adaptive interaction. This puts forward application of passive BCIs in neuroergonomic HRI (Lotte and Roy, 2019) where potential mental overload, attention drops, negative emotions, and human errors can be prevented or managed in an online and unobtrusive manner, thereby increasing the interactivity between the user and the robot and facilitating their collaboration (Krol et al., 2018). Meanwhile, more research is required in the field of HRI to formulate appropriate design principles for context-aware alignment of the robot behavior with human expectations, needs and conventions, once such higher order information from the user is available (Rossi et al., 2017; Sciutti et al., 2018).

Another direction toward future collaboration between passive BCI and HRI research could be development of social robots that assist neurofeedback training for augmented cognition or sustenance of a desirable psychological state (Anzalone et al., 2016; Alimardani and Hiraki, 2017; Alimardani et al., 2018, 2020; Tsiakas et al., 2018; Cinel et al., 2019). One of the main problems with the traditional neurofeedback training paradigms is that the changes in brain features are usually presented to the users through auditory or visual feedback. This lacks engagement with the interface, which makes the training after a short while tedious. Recent works have replaced the old protocol with interactive computer games (Mishra et al., 2016) and immersive virtual environments (Kosunen et al., 2016). However, these applications require steady visual attention toward a computer screen or placement of a head-mounted display over the EEG electrodes that can be intrusive to the user and cause cybersickness. A social robot on the other hand, induces a feeling of co-presence, mind perception, and emotional support (Alimardani and Qurashi, 2019), which can positively influence performance, motivation, and social interaction during a training program (Wiese et al., 2017; Sinnema and Alimardani, 2019; Alimardani et al., 2020). Past research has shown that the physical embodiment of an agent generates a more natural, efficient, and joyful communication during elderly cognitive training (Tapus et al., 2009) as well as a higher learning gain during tutoring interactions (Leyzberg et al., 2012). Therefore, it is expected that a robot-guided cognitive training would extend similar benefits compared to previous non-social environments (Pino et al., 2019).

Although passive BCIs provide substantial opportunity for optimization of performance and interactivity in HRI, their advantages are often mitigated by several limitations with respect to real-world implementation. One of the general challenges in the usage of BCIs in real-world conditions is the high cost and long preparation time that is required for the hardware setup (electrode placement) and software tuning (individualized calibration). Recent development of wireless EEG caps and low-cost commercial headsets has substantially reduced the setup time for real-world recordings, however they often come at the cost of precision and reliability. Also, there have been attempts in reducing calibration time by means of machine learning techniques and adaptive classifiers that extract common features among all users (Lotte, 2015), known as inter-subject associativity (Saha and Baumert, 2019). On the other hand, deep learning methods have been suggested for automatic learning of representations in the brain activity, thereby reducing the pre-processing and manual feature extraction that is required for BCI classifier training (Nagel and Spüler, 2019; Tanveer et al., 2019). For BCI technology to become mainstream and be employed by non-experts in other research domains, we must reduce the cost of equipment use while improving the quality of recording and precision of algorithms. Hence, further advancement in wearable sensor technology as well as progress in signal processing techniques and computational modeling of brain activity is required for the BCIs to be finally deployed in every-day use.

Another constraint in employing BCIs in real-world scenarios is vulnerability of BCI output to external noise (Minguillon et al., 2017). In most BCI studies, participants are instructed to relax during the recording and avoid unnecessary movements; nevertheless the online performance of these systems is yet far from ideal due to uncontrolled concomitant stimulus in the environment and diverse neurophysiological dynamics across individuals. In the case of passive BCIs, this is an even more severe issue as the user's involvement in another task or integration of the BCI system with other types of technology introduces new artifacts from the environment resulting in undesirable outcome (Zander et al., 2010). Such misclassifications can become particularly critical in the HRI scenarios, as poor performance from the system will produce unwanted behavior from the robot, thereby harming the interaction quality and diminishing the expected effects. A proposed solution for this problem is combination of multiple brain imaging modalities, such as fNIRS and EEG, to develop hybrid BCIs that benefit from both high temporal and high spatial resolution and hence can provide better accuracy and process more commands from the user (Hong and Khan, 2017; Dehais et al., 2018). Similarly, combination of brain signals with other physiological data such as electromyography (EMG) or electrooculography (EOG) can help detect and reduce the effect of noise and increase the number of control commands necessary for multi-task control (Hong and Khan, 2017; Zhang et al., 2019).

In the same vein, care must be taken when collecting data for development of passive BCIs models in complex environments where alternative sources of cognitive and affective stimuli are available. Mappings between target mental states and brain activity should clearly be defined and investigated with careful consideration of confounding factors that might affect neurophysiological variables (Brouwer et al., 2015). For instance, when developing an affective BCI classifier for detection of human emotions during interaction with a robot, the BCI model should be trained and tested in an ecologically valid HRI setting rather than with a set of affective visual stimuli. Such new experimental paradigms may lead to unsuccessful or inconsistent results compared to prior neuroscience studies, however, this should not demotivate researchers from reporting their findings as the BCI field is still in its infancy and the report of negative results is equally valuable for its further progress (Lotte et al., 2020).

Yet, another challenge with respect to integration of passive BCIs in human-robot interaction studies is the high demand for computational resources and data storage, which are indispensible to real-time processing of brain activity as well as real-time configuration of the robot controller. This means that in practice, the two interfaces are often operated on different computers/environments and hence need to communicate with one another through proxy solutions (Müller-Putz et al., 2011). In order to integrate BCIs and robots efficiently, future developments is required to provide cost-effective BCI modules that can be compiled and implemented in multiple environments without requiring extensive programming and adaptation.

Last but not least, we should not lose sight on the emerging ethical issues in real-world employment of passive BCIs such as management of user expectation and sensitive data (Burwell et al., 2017). Obviously, the idea of continuous monitoring and access to someone's thoughts is dreadful, particularly when this information is collected and processed by a humanlike entity such as a robot. Especially, in the case of affective BCIs, there are unique challenges with respect to user autonomy as they entail the risk of manipulation or inducement of affective states without the user's consent (Steinert and Friedrich, 2020). Therefore, it is of high importance to scrutinize the ethical implications of BCI-driven robots and develop educational programs that communicate ethical guidelines to potential users before such technologies are released into the wild.

## Conclusion

Passive BCI technology holds promise in extracting affective and cognitive states for an optimized human-technology interaction. In this paper, we laid out the current state of the art in passive BCIs and illustrated their implications for real-world applications. We particularly reviewed their possible employment in human-robot interaction with the intention to inform the HRI community of the promises and challenges of passive BCI technology. Future work should continue to advance the synergy between the two domains and further explore the impact and effectiveness of BCI-driven robots during closed-loop interactions with humans.

## Author Contributions

MA wrote the manuscript with subsequent input from KH. All authors contributed to the article and approved the submitted version.

## Conflict of Interest

The authors declare that the research was conducted in the absence of any commercial or financial relationships that could be construed as a potential conflict of interest.
